# Editorial: Unraveling protein misfolding and innate immunity in neurodegenerative diseases

**DOI:** 10.3389/fimmu.2026.1873694

**Published:** 2026-05-28

**Authors:** Qianqian Fang, Yinghui Hong, Biyue Zhu

**Affiliations:** 1 Department of Pharmacy Children’s Hospital of Chongqing Medical University, National Clinical Research Center for Children and Adolescents’ Health and Diseases, Ministry of Education Key Laboratory of Child Development and Disorders, International Science and Technology Cooperation Base of Child Development and Critical Disorders, Chongqing Key Laboratory of Child, Neurodevelopment and Cognitive Disorders, Intelligent Application of Big Data in Pediatrics Engineering Research Center of Chongqing Education Commission of China, Chongqing, China

**Keywords:** artificial intelligence, innate immunity, neurodegenerative diseases, neuroinflammation, protein misfolding

Protein misfolding and aggregation stand as important pathological features of neurodegenerative diseases (NDs), such as Alzheimer’s disease, Parkinson’s disease (PD), and amyotrophic lateral sclerosis (ALS). Protein misfolding and aggregation drive both neuronal injury and illness progression (1, 2). The innate immune system is a vital component of the brain’s defensive machinery. It plays a dual role in NDs—on one hand helping to clear abnormal proteins, on the other hand potentially fueling neuroinflammation and worsening pathology (3, 4). Research in this area is expanding. However, the interaction between protein misfolding and innate immunity remains elusive. Deeper investigation is urgently needed. This Research Topic helps decode these interactions. It offers new views on ND pathogenesis and possible treatments.

The four articles collected in this Research Topic collectively deepen our comprehension of the connections among protein misfolding, innate immunity, and clinical disease burden, spanning cellular mechanisms, AI-driven approaches, and clinical immunophenotyping. Regarding environmental triggers of proteinopathy, Sini et al. show through cell-based experiments that the cyanotoxin L-BMAA disrupts autophagic flux, leading to intracellular accumulation of α-synuclein and TDP-43 with aberrant cytoplasmic localization (5). This study provides the first direct experimental evidence that a cyanotoxin can drive two distinct proteinopathies by interfering with proteostasis. From a computational perspective, Deng et al. offer a comprehensive analysis of recent advances in artificial intelligence, illustrating how AI can be used to predict the conformational traits of misfolded proteins, simulate the dynamics of protein aggregation, uncover how the innate immune system recognizes these abnormal proteins, and reconstruct the regulatory networks governing neuroinflammation (6). Their work elegantly bridges structural bioinformatics and neuroimmunology across both adult and pediatric neurodegenerative diseases. On the clinical side, Ji et al. conducted a multicenter cohort study on neuronal intranuclear inclusion disease (NIID) and identified a persistent innate immune signature—elevated levels of neutrophils, monocytes, and IL-6—that associates with kidney injury independently of GGC repeat expansion size (7). This study provides clinical evidence that acquired inflammatory mechanisms determine the renal phenotype in a repeat-expansion proteinopathy. Complementing these mechanistic and clinical investigations, Fang et al. used a machine learning-based bibliometric method to analyze 9,277 publications on Duchenne muscular dystrophy, mapping the global research hotspots and evolving trends over two decades (8). Their analysis highlights essential areas encompassing disease mechanisms, diagnostic approaches, and treatment strategies, thereby offering a valuable foundation for advancing fundamental research and refining clinical practice.

Together, these findings point to a common theme. Abnormal protein aggregates are not just disease byproducts. They actively shape innate immune responses. Whether triggered by L-BMAA or a genetic repeat expansion, protein misfolding impairs autophagy, activates stress pathways, and raises IL-6 levels. That remodels the immune microenvironment. Still, questions remain. We do not know which pattern recognition receptors recognize different misfolded forms. We also do not know if the immune response helps or hurts over time. Ji et al. noted that kidney injury in NIID does not simply track with repeat expansion size (7). So environmental factors probably matter. How they work is unclear. In future considerations, boosting autophagy could be a therapy, as Sini et al. showed. The cyanobacteria-gut-brain axis is another area to explore (5). Clinically, prospective cohort studies should test if NLR and IL-6 can serve as early markers of organ damage. Safe therapeutic windows are also needed—targeting IL-6 without compromising systemic immunity. Deng et al. reviewed how AI-based modeling can predict which protein epitopes trigger immune responses, guiding immunomodulation (6). Fang et al. mapped DMD research and found that inflammation, oxidative stress, and multidisciplinary care are key themes (8). These efforts could shift the field from clearing protein clumps to targeting protein-immune crosstalk, opening new paths for treating neurodegenerative disorders.

We sincerely thank all authors for their valuable contributions to this Research Topic. They have greatly enriched our understanding of NDs. We encourage readers to delve into the full texts for a more comprehensive perspective. We hope that this Research Topic will inspire further research and foster continued progress in the fight against these devastating diseases.
